# An *in silico *analysis of dynamic changes in microRNA expression profiles in stepwise development of nasopharyngeal carcinoma

**DOI:** 10.1186/1755-8794-5-3

**Published:** 2012-01-19

**Authors:** Zhaohui Luo, Liyang Zhang, Zheng Li, Xiayu Li, Gang Li, Haibo Yu, Chen Jiang, Yafei Dai, Xiaofang Guo, Juanjuan Xiang, Guiyuan Li

**Affiliations:** 1 Cancer Research Institute, Key Laboratory of Carcinogenesis and Cancer Invasion of Ministry of Education, Key Laboratory of Carcinogenesis of Ministry of Health, Central South University, 110 Xiangya Road, Changsha, Hunan, 410078, P.R. China; 2 The Li Ka Shing Institute of Health Sciences, Department of Orthopaedics & Traumatology, Faculty of Medicine, The Chinese University of Hong Kong, Prince of Wales Hospital, Shatin, NT, Hong Kong

## Abstract

**Background:**

MicroRNAs (miRNAs) are small non-coding RNAs that participate in the spatiotemporal regulation of messenger RNA (mRNA) and protein synthesis. Recent studies have shown that some miRNAs are involved in the progression of nasopharyngeal carcinoma (NPC). However, the aberrant miRNAs implicated in different clinical stages of NPC remain unknown and their functions have not been systematically studied.

**Methods:**

In this study, miRNA microarray assay was performed on biopsies from different clinical stages of NPC. TargetScan was used to predict the target genes of the miRNAs. The target gene list was narrowed down by searching the data from the UniGene database to identify the nasopharyngeal-specific genes. The data reduction strategy was used to overlay with nasopharyngeal-specifically expressed miRNA target genes and complementary DNA (cDNA) expression data. The selected target genes were analyzed in the Gene Ontology (GO) biological process and Kyoto Encyclopedia of Genes and Genomes (KEGG) biological pathway. The microRNA-Gene-Network was build based on the interactions of miRNAs and target genes. miRNA promoters were analyzed for the transcription factor (TF) binding sites. UCSC Genome database was used to construct the TF-miRNAs interaction networks.

**Results:**

Forty-eight miRNAs with significant change were obtained by Multi-Class Dif. The most enriched GO terms in the predicted target genes of miRNA were cell proliferation, cell migration and cell matrix adhesion. KEGG analysis showed that target genes were significantly involved in adherens junction, cell adhesion molecules, p53 signalling pathway et al. Comprehensive analysis of the coordinate expression of miRNAs and mRNAs reveals that miR-29a/c, miR-34b, miR-34c-3p, miR-34c-5p, miR-429, miR-203, miR-222, miR-1/206, miR-141, miR-18a/b, miR-544, miR-205 and miR-149 may play important roles on the development of NPC. We proposed an integrative strategy for identifying the miRNA-mRNA regulatory modules and TF-miRNA regulatory networks. TF including ETS2, MYB, Sp1, KLF6, NFE2, PCBP1 and TMEM54 exert regulatory functions on the miRNA expression.

**Conclusions:**

This study provides perspective on the microRNA expression during the development of NPC. It revealed the global trends in miRNA interactome in NPC. It concluded that miRNAs might play important regulatory roles through the target genes and transcription factors in the stepwise development of NPC.

## Background

Genetic and environmental factors are involved in the tumorigenesis and development of nasopharyngeal carcinoma (NPC). NPC is commonly diagnosed late due to vague early symptoms [1]. At first consultation, 70% of cases were diagnosed as cervical lymph node metastasis and 20-35% were diagnosed as long distant metastasis [2-4]. It is important to elucidate the cellular and molecular mechanisms of dynamic development of NPC. It is extremely necessary to identify the biomarkers and detect the high-risk factors in the progression of NPC.

microRNAs (or 'miRNAs', which are small noncoding RNA molecules) as post-transcriptional regulators have been a hotspot in research for their involvement in biological processes and tumour development [5,6]. They have been found to regulate genes involved in diverse biological functions, including development, differentiation, proliferation, and stress response. The dysregulation of miRNAs appears to play a crucial role in cancer pathogenesis where they exert their effect as oncogenes or as tumour suppressors [7]. A growing number of miRNAs have been implicated in carcinogenic process. A significant number of miRNAs have been mapped to cancer-associated genomic regions. Expression of the miRNA let-7 has been correlated with prognosis in lung cancer and found to regulate Ras in the same tumor [8]. Very recently, miR-10b has been shown to contribute to metastasis in breast cancer [9].

To date, several miRNAs have been shown to target specific mRNAs to regulate the progression of NPC. miR-216b [10], miR-218 [11], miR-26a/b [12,13], miR-10b [14], let-7 [15], miR-141 [16], miR-200a [17] have been shown to have tumor suppressive functions in NPC. Not surprisingly, Epstein-Barr virus-encoded miRNAs have oncogenic properties [18-20]. It is well known that EBV infection has been identified as an essential factor in the carcinogenesis of NPC [21]. EBV-infection severely deregulates the miRNA profile of the host cell [22]. Several EBV encoded miRNAs were expressed at levels similar to highly abundant human miRNAs. EBV-encoded miRNAs such as miR-BART1-5p, miR-BART16, and miRBART17-5p, are intimately involved in processes leading to NPC [23,24]. The microRNA array has been performed by independent labs, identifying several differentially-expressed miRNAs in NPC [25-27]. The development of NPC is a multistage process, depending on spatial and temporal control of gene expression. Thus, it is unclear whether dysregulation of microRNA expression is an aberrant event that occurs during NPC progression. The dynamic regulatory roles of miRNA need to be explored. A key to understanding the role of miRNA is to determine when and where they are expressed [28]. Here, we studied the miRNA dynamic expression profiles in different clinical stages of NPC and NPC lymph node metastasis. The predicted miRNA target genes were compared to the cDNA expression in the Gene Expression Omnibus (GEO) (GSE12452). We found that a series of genes and miRNAs play an important role in the stepwise development of NPC.

Similar to protein-coding genes, the transcription of miRNAs is also regulated by transcription factors (TFs) [29,30], an important class of gene regulators that act at the transcriptional level. The normal regulation of miRNAs by TFs is critical, and aberrant regulation of miRNAs by TFs can cause phenotypic variations and diseases [31]. Therefore, a TF-miRNA regulation database would be helpful for understanding the mechanisms by which TFs regulate miRNAs and then understanding their contribution to diseases.

In this study, the miRNA-gene interaction and transcription factor-miRNA interaction network were also described. Our study gives perspective on miRNAs expression in the stepwise development of NPC. It revealed the global trends in miRNA interactome in NPC.

## Methods

### Patient samples and laser-capture microdissection

Snap-frozen NPC biopsies were obtained from NPC patients and normal, healthy nasopharyngeal epithelial samples from biopsy-negative cases were used as control. The criteria of clinical staging of NPC samples was based on the 2008 staging system of NPC, which was established in 2008 according to NPC 92 and AJCC staging system [32,33]. NPC samples in clinical staging I-IV were used (numbers I to IV, with IV having greater progression). Samples were collected from Xiangya Second Hospital, Central South University. The patients were informed about the sample collection and had signed informed consent forms. Collections and use of tissue samples were approved by the ethical review committees of Xiangya Second Hospital (Additional file 1). Laser capture microdissection was used to separate the cancer tissues from the normal tissues. Samples were first frozen-sectioned by using a LEICA CM 1900 cryomicrotome. Phase contrast images were acquired using LEICA CTR 6500 microscope.

### miRNA microarrays

Total RNA was extracted using Trizol^® ^reagent (Invitrogen) from samples. Two hundred nanograms (200 ng) of total RNA from each sample were used for the follow-up microarray. Poly(A) polymerase (PAP) was used to add a stretch of Poly-A tail to the 3' end of each sequence in total RNA. The Ambion Illumina TotalPrep RNA Amplification Kit was used to synthesize biotinylated cDNA. MicroRNA expression profiling kit contains primers for 1146 human miRNAs. The biotinylated cDNAs were hybridized with microRNA-specific oligonucleotides. The unbinding oligos were washed away and followed by the extension and ligation reaction. Polymerase Chain Reactions (PCR) were performed with fluorescently labelled universal primers, followed by hybridizing of the fluorescently labelled, single-stranded PCR products to capture beads. The fluorescent signals were then detected by Illumina's iScan System. All steps were performed according to Illumina's instructions manual.

### Bioinformatics analysis

#### 1. Multi-Class Dif (RVM-F test)

Raw data from each array were analyzed using Multi-Class Dif (RVM-F test) which is applicable to small sample size analysis for multiple groups. The RVM F-test was applied to screen the dynamic differentially-expressed genes. The detailed methods were applied as previously described [34-36].

#### 2. Gene Ontology (GO)

The TargetScan database was used to predict the target gene of miRNAs. To understand the functions of predicted target genes, we used the ontology classification of genes based on gene annotation and summary information available through DAVID (Database for Annotation, Visualization and Integrated Discovery). The predicted target genes were assigned to functional groups based on molecular function, biological processes and specific pathways.

#### 3. TF-miR-net

miRNAs sequence was mapped to the genome in the Sanger database. The Jemboss software was used to examine the alignment of sequences of pre-miRs and putative transcription factor binding sequences. A genome browser database was used to build the relationship of transcription factors and miRNAs network. An adjacency matrix was implemented in Java (programming language) according to the binding of pre-miRNAs and transcription factors. The network's core transcription factor is the most important centre with the biggest degree [37,38]. The Pearson correlation analysis [37] is used to measure the regulatory ability of transcription factors by calculating the correlation between transcription factors and the miRNAs.

#### 4. MicroRNA-gene-network

The MicroRNA-Gene-Network was based on the interactions of miRNAs and target genes [39]. Twenty miRNAs of interest were built determined by pathways extracted from KEGG as primary nodes-networks. The significance of relationship of the miRNAs and target genes network was evaluated by the number of nodes in the network with degree greater than 10. In the MicroRNA-Gene-Network, the circle represents gene and the square represents MicroRNA, and their relationship was represented by one edge.

### Quantitative reverse transcription-polymerase chain reaction analysis

Total RNA was extracted using Trizol^® ^reagent (Invitrogen) from samples. The primers for RT-PCR to detect miRNA were designed based on the miRNA sequences provided by the Sanger Center miRNA Registry. The primers were synthesized and purified by the Shanghai Gene-Pharma Co. (Shanghai, China). RT reactions were performed by means of the iScript cDNA synthesis kit (Bio-Rad, Hercules, CA). Real-time PCR was performed on the BIO-RAD IQTM5 Multicolor Real-Time PCR detection System (Bio-Rad). The qPCR cycle was 98°C for 2 min., 40 cycles of 95°C for 15 sec., 60°C for 30 sec. Final melt-curve analysis (60°-95°C) was included. The standard curve was produced with slopes at approximately -3.32 (~100% efficiency); miRNA PCR quantification used 2^ΔΔct ^method against the U6 for normalization. mRNA PCR quantification used 2^ΔΔct ^method against the GAPDH for normalization. The data are representative of the means of three experiments.

RT-PCR primers:

miR-18a-F: TAAGGTGCATCTAGTGCAGATAG

miR-18b-F: TAAGGTGCATCTAGTGCAGTTAG

mR-141-F: TAACACTGTCTGGTAAAGATGG

miR-149-F: TCTGGCTCCGTGTCTTCACTCCC

miR-99a-F: AACCCGTAGATCCGATCTTGTG

miR-99b-F: CACCCGTAGAACCGACCTTGCG

miR-206-F: TGGAATGTAAGGAAGTGTGTGG

miR-34b-F: CAATCACTAACTCCACTGCCAT

miR-34c-3-F: AATCACTAACCACACGGCCAGG

miR-34c-5-F: AGGCAGTGTAGTTAGCTGATTGC

miR-29b-F: TAGCACCATTTGAAATCAGTGTT

miR-29c-F: TAGCACCATTTGAAATCGGTTA

miR-429-F: TAATACTGTCTGGTAAAACCGT

miR-32-F: TATTGCACATTACTAAGTTGCA

miR-181c-F: AACATTCAACCTGTCGGTGAGT

U6-F: ATTGGAACGATACAGAGAAGATT

U6-R: GGAACGCTTCACGAATTTG

ATM-F: GGACAGTGGAGGCACAAAAT

ATM-R: GTGTCGAAGACAGCTGGTGA

BCL2-L2-F: TCGCCCTGTGGATGACTGA

BCL2-L2-R: CCAGGAGAAATCAAACAGAGGC

CDH1-F: CGAGAGCTACACGTTCACGG

CDH1-R: CGAGAGCTACACGTTCACGG

E2F3-F: CACTTCCACCACCTCCTGTT

E2F3-R: TGACCGCTTTCTCCTAGCTC

YY1-F: CAAGAAGTGGGAGCAGAAGC

YY1-R: CTGCCAGTTGTTTGGGATCT

MYB-F: ACAGTCATTTGATGGGTT

MYB-Re TCTCGGTTGACATTAGGA

Dcier-Fe AAGGAAGCTGGCAAACAAGAe

Dcier-R: AAAACGAACCACCAAGTTGCe

Smad2-F: CGAAATGCCACGGTAGAAAT

Smad2-R: CCAGAAGAGCAGCAAATTCC

KLF6-F: CACGAGACCGGCTACTTCTC

KLF6-R: CGGATTCCTCCTTTTTCTCC

GAPDH-F: GAGTCAACGGATTTGGTCGT

GAPDH-R: TTGATTTTGGAGGGATCTCG

## Results

### 1. miRNA expression profile in the stepwise development of NPC

**Figure 1:**
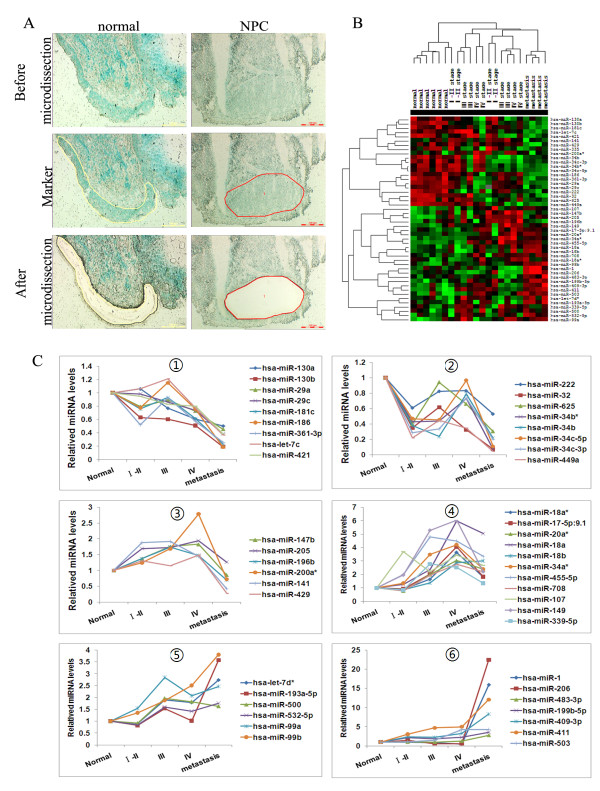
**miRNA expression profile in the stepwise development of NPC**. (A) The microdissection was performed with Methyl Green staining to separate tumor cells to non-tumor cells; (B)Hierarchical clustering result of 48 differentially-expressed miRNAs. Each row represents the expression profile of a miRNA across 22 samples and each column represents a sample. The sample IDs and clinical stages information are listed above the 'heatmap'. Red and green colors respectively indicate either higher and lower expression levels of the miRNA. Samples are well separated into control and NPC patient groups except three. Using the dendrogram-based methods for clustering, the samples can be further separated into five subgroups on hierarchical clustering, which were correlated with the NPC clinic stages. (C) Dynamic miRNA expressions were classified into 6 different patterns.

In order to identify miRNAs associated with the stepwise development of NPC, miRNA microarrays were performed. We analyzed the temporal patterns of miRNAs expression profiles during the development of NPC. Samples were taken from a range of tumors of different stages. 6 cases of normal, 4 cases of each stage I or II, III, IV and 4 cases of lymph node metastasis were taken. The microdissection was performed with Methyl Green staining to separate tumor cells to non-tumor cells (Figure 1A). The Illumina microRNA Expression Profiling Assay was performed. The raw data of miRNA array can be downloaded from the National Center for Biotechnology Information-Gene Expression Omnibus (GEO) (GEO:GSE32906). From MicroRNA profiling, after separating signal from noise, we obtained 766 miRNAs. Microarray data showed that 48 miRNAs were differentially expressed with significant change in tumor samples compared to normal samples (Multi-class Dif multiple comparison test RVM-F test, P < 0.05, FDR < 0.05) [34-36]. Differentially expressed miRNAs between various stages of NPC and normal nasopharyngeal epithelia were clustered by Cluster3.0, as shown in the Figure 1B. Using the dendrogram-based methods for Clustering, the samples can be further separated into five subgroups on hierarchical clustering based on their similar expression patterns, which were correlated with the NPC clinic stages. The results showed that the expression pattern of miRNAs from the different clinical stages can be distinguished from each other (Figure 1B). We identified differentially-expressed miRNAs between clinical stages, in which 12 miRNAs differentially expressed between stage I-II and normal, 15 miRNAs between stage III and normal, 20 miRNAs between stage IV and normal, 37 miRNAs between lymph node metastasis and normal, as shown in Table 1. Our study revealed dynamic miRNA expressions, which were classified into 6 different patterns (Figure 1C). In pattern 1, miRNA expressions gradually decreased during the development of NPC. In pattern 5, miRNA expressions gradually increased during the development of NPC. In pattern 6, miRNA expressions dramatically increased in the lymph node metastasis.

**Table 1:** The aberrantly expressed miRNAs in the different multi-stages of NPC

Stage	Down-regulated miRNAs	Up-regulated miRNAs
I-II	hsa-miR-449a, hsa-miR-34c-3p, hsa-miR-32, hsa-miR-34b, hsa-miR-625, hsa-miR-34b* hsa-miR-34c-5p	hsa-miR-455-5p, hsa-miR-199b-5p, hsa-miR-409-3p, hsa-miR-411, hsa-miR-107
III	hsa-miR-34b, hsa-miR-34c-3p, hsa-miR-34b*, hsa-miR-449a, hsa-miR-34c-5p	hsa-miR-17-5p:9.1, hsa-miR-107, hsa-miR-409-3p, hsa-miR-18a, hsa-miR-339-5p, hsa-miR-99a, hsa-miR-34a*, hsa-miR-411, hsa-miR-455-5p, hsa-miR-149
IV	hsa-miR-32, hsa-miR-449a,	hsa-miR-99a, hsa-miR-199b-5p, hsa-miR-99b, hsa-miR-339-5p, hsa-miR-708, hsa-miR-200a*, hsa-miR-18b, hsa-miR-20a*, hsa-miR-409-3p, hsa-miR-107, hsa-miR-18a*, hsa-miR-17-5p:9.1, hsa-miR-34a*, hsa-miR-503, hsa-miR-455-5p, hsa-miR-411, hsa-miR-18a, hsa-miR-149
Metastasis	hsa-miR-449a, hsa-miR-32, hsa-miR-34b*, hsa-miR-34c-5p, hsa-miR-186, hsa-miR-130b, hsa-miR-34c-3p, hsa-miR-34b, hsa-miR-181c, hsa-miR-361-3p, hsa-miR-429, hsa-miR-625, hsa-miR-335, hsa-let-7c, hsa-miR-29c, hsa-miR-421, hsa-miR-141, hsa-miR-29a	hsa-miR-708, hsa-miR-149, hsa-miR-20a*, hsa-miR-34a*, hsa-miR-99a, hsa-miR-107, hsa-let-7d*, hsa-miR-483-3p, hsa-miR-18b, hsa-miR-455-5p, hsa-miR-199b-5p, hsa-miR-193a-5p, hsa-miR-99b, hsa-miR-503, hsa-miR-18a, hsa-miR-409-3p, hsa-miR-411, hsa-miR-1, hsa-miR-206

These results suggested that these miRNAs might play important roles in the stepwise development of NPC.

### 2. Target gene prediction of miRNAs and gene function analysis

Putative target genes of 48 differentially expressed miRNAs were searched with online algorithms for miRNA target prediction (TargetScan). More than a thousand target genes were predicted for the 48 miRNAs. The predicted target gene lists were narrowed down by searching the data from ftp://ftp.ncbi.nih.gov/repository/UniGene/ to find the nasopharyngeal- specific genes.

**Figure 2:**
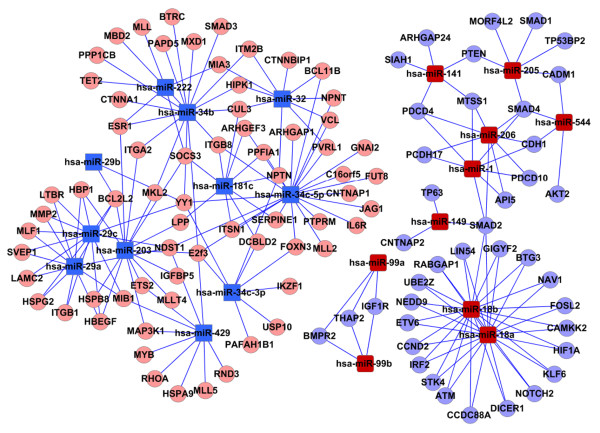
**Target gene prediction of miRNAs**. miRNA-gene interactions were built into a bipartite network (the miRNAome). The red rectangles indicate the up-regulated miRNAs, the blue rectangles indicate the down-regulated miRNAs. The red ellipses indicate the up-regulated target genes, the blue ellipses indicate the down-regulated target genes.

We compared gene expression profiles of NPC biopies and miRNA expression profiles done above. The expression data (GSE12452) from biopsies of NPC and non-malignant controls were downloaded from the National Center for Biotechnology Information-Gene Expression Omnibus (GEO). Putative target genes of miRNAs differentially expressed in NPC biopsies, which was consistent with the miRNA expression were shown in Table 2. We found that such as miR-29c and miR-34c-5 were down regulated, their target gene NDST1 and MMP2 were upregulated in NPC, miR-1/206 and their target CDH1, SMAD4, PDCD10, TGFBR3 are also consistent to each other, miR-18a/b and their target ATM and Samd4 also showed the coordinate expression. After microRNAs target filtering, the miRNAs which regulate the development of NPC through target gene regulation may be narrowed down. The selected genes were analyzed in the context of Gene Ontology (GO) biological process and Kyoto Encyclopedia of Genes and Genomes (KEGG) biological pathway using the molecular annotation. To assess the function of the predicted target gene, we evaluated the frequency of specific gene ontology terms among the predicted nasopharyngeal specific target genes of the 48 miRNAs using DAVID. The most enriched GO terms in the predicted targets genes of miRNA were cell proliferation, cell migration and cell matrix adhesion (Table 3). The predicted target genes were also analyzed by Kyoto Encyclopedia of Genes and Genomes (KEGG). As shown in Table 4, these target genes were significantly involved in adherens junction, pathway in cancer, cell adhesion molecules, p53 signaling pathway *et al*. It demonstrated that miRNAs with significant change are involved in regulation of target genes related to the development of NPC. These miRNA:gene interactions were built into a bipartite network (the miRNAome) (Figure 2).

**Table 2:** Putative target genes of differentially expressed miRNAs in NPC biopsies, which was consistent with the mRNA expression

miRNA microarray		cDNA microarray	
miRNA	Expression	Target gene	expression
hsa-miR-1/206	up	API5, PCDH17, SMAD2, MTSS1, PDCD10, PDCD4, SMAD4	down
hsa-miR-141	up	PDCD4, MTSS1, ARHGAP24, SIAH1, PTEN	down
hsa-miR-149	up	TP63, SMAD2, CNTNAP2	down
hsa-miR-18a/b	up	DICER1, GIGYF2, STK4, RABGAP1, NOTCH2, NEDD9, ATM, SMAD2, NAV1, FOSL2, CAMKK2, STK4, BTG3, UBE2Z, IRF2, LIN54, HIF1A, RABGAP1, CCDC88A, ETV6, CCND2	down
hsa-miR-205	up	CADM1, MORF4L2, PTEN, SMAD1, SMAD4, TP53BP2	down
hsa-miR-544	up	AKT2, CADM1, CDH1, SMAD4	down
hsa-miR-99a/b	up	BMPR2, IGF1R, THAP2	down
hsa-miR-203	down	BCL2L2, ESR1, HBEGF, HBP1, HSPB8, IGFBP5, ITGA2, LPP, MAP3K1 MKL2, MLLT4, SOCS3	up
hsa-miR-222	down	ESR1, ITGB8, MBD2, MIA3, MLL, SOCS3, TET2	up
hsa-miR-29a/c	down	BCL2L2, HBEGF, HBP1, HSPG2, ITGB1, LAMC2, LTBR, MIB1, MLF1, MMP2, NDST1, SVEP1	up
hsa-miR-34b	down	BTRC, CTNNA1, CUL3, CYLD, ESR1, HIPK1, ITGA2, ITM2B, MKL2, MXD1, PAPD5, PPP1CB, SMAD3	up
hsa-miR-34c-3p	down	DCBLD2, FOXN3, IKZF1, NPTN PAFAH1B1, USP10, YY1	up
hsa-miR-34c-5p	down	ARHGAP1, ARHGEF3, BCL11B, C16orf5, CNTNAP1, FOXN3, FUT8, IL6R, ITGB8, ITSN1, JAG1, MLL2, NDST1, NOTCH2, NPNT, PPFIA1, PTPRM, PVRL1, SERPINE1, VCL, YY1	up
hsa-miR-429	down	MIB1, MLL5, NDST1, RHOA, RND3	up

**Table 3:** GO Functional Annotation of the target genes

Term	Count	%	PValue	FDR
up-regulated target gene GO functional annotation				
GO:0022610~biological adhesion	19	2.496715	2.93E-09	4.71E-06
GO:0007155~cell adhesion	19	2.496715	2.87E-09	4.60E-06
GO:0010941~regulation of cell death	17	2.233903	1.04E-06	0.00166506
GO:0043067~regulation of programmed cell death	17	2.233903	9.88E-07	0.001585717
GO:0042981~regulation of apoptosis	17	2.233903	8.67E-07	0.0013906
GO:0042127~regulation of cell proliferation	17	2.233903	6.52E-07	0.001046319
GO:0007049~cell cycle	16	2.102497	2.94E-06	0.00471089
GO:0051270~regulation of cell motion	10	1.31406	3.26E-07	5.23E-04
GO:0040012~regulation of locomotion	10	1.31406	3.12E-07	5.01E-04
GO:0030334~regulation of cell migration	10	1.31406	1.05E-07	1.69E-04
down-regulated target gene GO functional annotation				
GO:0042127~regulation of cell proliferation	13	2.901786	4.73E-07	7.26E-04
GO:0016265~death	12	2.678571	1.68E-06	0.00257469
GO:0008219~cell death	12	2.678571	1.57E-06	0.002405587
GO:0012501~programmed cell death	11	2.455357	2.87E-06	0.004409001
GO:0006915~apoptosis	11	2.455357	2.52E-06	0.003858936

**Table 4:** The KEGG-PATHWAY analysis of the target genes

Term	Count	%	PValue
up-regulated target gene KEGG-PATHWAY			
hsa04520:Adherens junction	7	0.9198423	5.40E-06
hsa04510:Focal adhesion	7	0.9198423	0.001138399
hsa04512:ECM-receptor interaction	5	0.6570302	0.001552176
hsa04514:Cell adhesion molecules (CAMs)	5	0.6570302	0.007927108
hsa04810:Regulation of actin cytoskeleton	6	0.7884363	0.008917732
hsa05200:Pathways in cancer	7	0.9198423	0.012815044
hsa04530:Tight junction	4	0.5256242	0.047009212
hsa04120:Ubiquitin mediated proteolysis	4	0.5256242	0.049654282
down-regulated target gene KEGG-PATHWAY			
hsa05200:Pathways in cancer	8	1.7857143	1.12E-04
hsa04115:p53 signaling pathway	4	0.8928571	0.001901548
hsa04520:Adherens junction	4	0.8928571	0.002717119
hsa05210:Colorectal cancer	4	0.8928571	0.003481217
hsa04350:TGF-beta signaling pathway	4	0.8928571	0.003845193
hsa04110:Cell cycle	4	0.8928571	0.010537531
hsa04310:Wnt signaling pathway	4	0.8928571	0.017542982
hsa04510:Focal adhesion	4	0.8928571	0.036959365

### 3. Validation of expression of microRNA and their target genes

**Figure 3:**
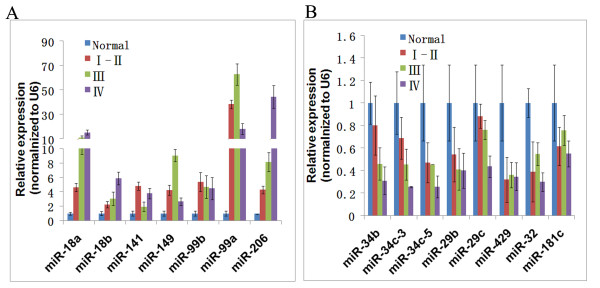
**The expression miRNAs were verified by real-time PCR in 38 NPC cases and 10 normal**. (A) the up-regulated miRNAs; (B) the down-regulated miRNAs.

**Figure 4:**
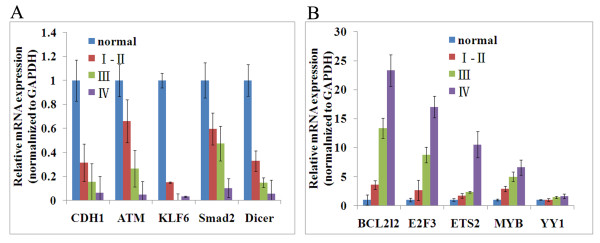
**The expression of corresponding target genes of miRNAs were verified by real-time PCR in 38 NPC cases and 10 normal**. (A) representative target genes of upregulated miRNAs; (B) representative target genes of downregulated miRNAs.

As mentioned above, we integrated the miRNA expression profile and cDNA expression profile. We narrowed down the candidate miRNA list which may play important roles on the development of NPC. To confirm microarray data, real-time RT-PCR experiments were conducted using specific primers for 15 miRNAs **(**miR-206, miR-141, miR-149, miR-34b, miR-34c-3, miR-34c-5, miR-18a, miR-18b, miR-99a, miR-99b, miR-429, miR-32, miR-181c, miR-29b and miR-29c) in 38 NPC cases and 10 normal nasopharyngeal epithelial tissue cases. We confirmed the down-regulation of the miR-34 family (miR-34b, miR-34c-3, miR-34c-5, miR-429) and up-regulation of miR-17-92 (miR-18a/b) cluster. The dynamic expression patterns obtained with real-time RT-PCR were consistent with the microarray results (Figure 3). Furthermore, we also performed the real-time PT-PCR to evaluate the expression target genes. As shown in Figure 4, the expression of the selected target genes of the miRNAs showed the reverse correlation with the miRNA expression. We found that target genes such as CDH1 (miR-1/206), ATM (miR-18a/b), KLF6 (miR-18a/b and miR-181c), Smad2(miR-18a/b, miR-1/206 and miR-149), Dicer(miR-18a/b) were down expressed with the development of NPC, while BCL2L2 (miR-29a/b/c and miR-203), E2F3(miR-34b/c and miR-429), ETS2(miR-429), MYB(miR-429) and YY1 (miR-29a/b/c and miR-34b/c) were overexpressed during the development of NPC.

### 4. The miRNA transcriptional network for NPC development

**Figure 5:**
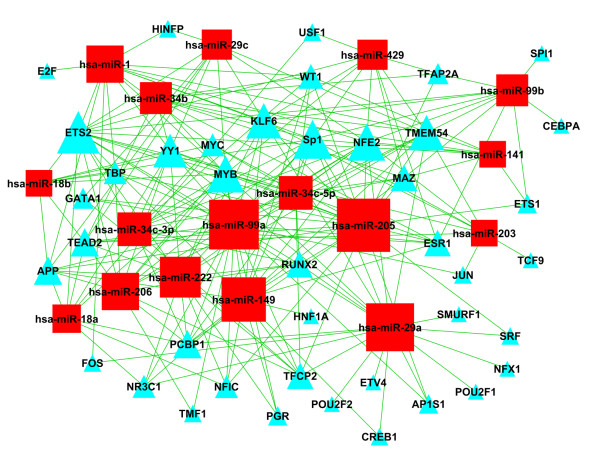
**The miRNA transcriptional network for NPC development**. These transcription factor-miRNA interactions were built into a network. The triangles represent transcription factors which regulate the miRNA expression; the squares represent the miRNAs which were regulated by TFs. The larger triangles represent the more miRNAs which are regulated.

Although the expression of miRNAs and their targets is often highly correlated, anti-correlations exist because of miRNA feedback loops and upstream regulators, including transcription factors. Except for the above miRNA-target genes network, the TF-miRNA transcriptional interactions need to be elucidated. The predicted transcription factors binding to the miRNAs with the degree greater than 10 were shown in Table 5. Among them, 7 transcription factors were found differentially expressed in clinical stage I-II; 8 in stage III; 14 in stage IV and 21 in lymph node metastasis, compared to the normal samples. Seven TFs including ETS2, MYB, Sp1, KLF6, NFE2, PCBP1 and TMEM54 were shown in the middle of network, regulating most of the selected miRNAs (Figure 5). The larger triangles represent the more miRNAs which are regulated. The numbers of miRNA regulated by TF were represented by degrees. These transcription factor-miRNA interactions were built into a network.

**Table 5:** The predicted transcription factors binding to the 48 aberrant miRNAs with the degree bigger than 10

Stage	degree ≥ 10
I-II	ETS2, MYB, Sp1, KLF6, NFE2, PCBP1, TMEM54
III	ETS2, MYB, KLF6, Sp1, PCBP1, TMEM54, YY1, NFE2
IV	ETS2, MYB, NFE2, PCBP1, APP, KLF6, Sp1, TMEM54, YY1, RUNX2, TFCP2, TEAD2, NR3C1, TFAP2A
Lymph node metastasis	ETS2, MYB, NFE2, Sp1, YY1, KLF6, TMEM54, ESR1, PCBP1, APP, TEAD2, RUNX2, TFCP2, TFAP2A, MAZ, NR3C1, TBP, WT1, MYC, AP1S1, USF1

We further investigated the TF-miRNAs-target genes feedback loop by integrating the TF-miRNA networks and miRNA-target genes networks. The possible regulatory feedback loops were shown in Table 6. These transcription factors may repress their own translation through induction of miRNAs as a negative regulatory feedback loop. This strategy combines paired expression profiles of TF, miRNAs and mRNAs in computational target predictions.

**Table 6:** TF-miRNAs-target genes feedback loop

TF	miRNA	target gene
ETS2	hsa-miR-199b-5p	ETS2
ETS2	hsa-miR-203	ETS2
ETS2	hsa-miR-222	ETS2
ETS2	hsa-miR-335	ETS2
ETS2	hsa-miR-429	ETS2
SP1	hsa-miR-1	SP1
SP1	hsa-miR-130a	SP1
SP1	hsa-miR-130b	SP1
SP1	hsa-miR-135a	SP1
SP1	hsa-miR-148b	SP1
SP1	hsa-miR-149	SP1
SP1	hsa-miR-181c	SP1
SP1	hsa-miR-199b-5p	SP1
SP1	hsa-miR-203	SP1
SP1	hsa-miR-206	SP1
SP1	hsa-miR-32	SP1
SP1	hsa-miR-335	SP1
SP1	hsa-miR-429	SP1
SP1	hsa-miR-486-3p	SP1
KLF6	hsa-miR-181c	KLF6

## Discussion

While the process events that result in NPC remain unclear, global transcriptome analysis, including miRNAome [40,41], is a useful tool to investigate dynamic change of molecular networks between different clinical stages. Although the microRNA array has been performed by two independent labs [25,26], the dynamic miRNA expression during the development of NPC remains unknown. NPC is a multistage process that usually takes decades to develop. It is necessary to investigate the molecular events during the process. In our study, samples from different clinical stages of NPC were analyzed by the microRNA array. Microdissection was performed to ensure the purity of cancer tissues. Usually microarray techniques provide a valuable way of characterizing the molecular nature of disease but the expense and limited specimen availability often lead to studies with small sample sizes. This makes accurate estimation of variability difficult. Since variance estimates made on a gene-by-gene basis will have few degrees of freedom, the assumption that all genes share equal variance is unlikely to be true. To solve this problem, in this study, Multi-Class Dif was used which is applicable to the small sample sizes and we found that 48 miRNAs were differentially expressed in the four development stages and lymph node metastasis.

In our study, we present a global relative expression analysis of miRNAs in NPC. miRNAs with similar expression profiles are clustered together in 6 patterns by cluster 3.0. In pattern 1, miRNA expressions gradually decreased during the development of NPC, which may function as a tumor suppressor gene, such as miR-29 [42,43]. In pattern 5, miRNA expressions gradually increased during the development of NPC, such as miR-18a/b. miR-18a/b was clustered together with miR-17-92 [44,45] according to the similar expression profiles, which were induced by c-myc and reported to promote cell differentiation and proliferation. In pattern 6, such as miR-206 [46] and miR-1 [47], miRNA expressions dramatically increased in the lymph node metastasis. It indicated that these miRNAs may play an important role in the metastasis and may be a special biomarker for the lymph node metastasis.

Bioinformatic algorithms have played a key role in the discovery of miRNAs, prediction of target genes and miRNAome. TargetScan, PicTar and miRanda were commonly used to predict miRNA target genes. We identified thousands of target genes in which only a small fraction of miRNA are actually involved in the biological process. The algorithms mentioned above showed limited consistency among predicted targets. It is necessary to identify the confirmed target genes which are actually related to the miRNA regulation function. In this study, the thousands of target genes were refined by screening with the nasopharyngeal- specifically expressed genes. Another data reduction strategy is to compare the miRNA expression and cDNA expression data and select the miRNA which is consistent with the cDNA expression [48]. The cDNA expression data (GSE12452) were downloaded from the publicly-accessible National Center for Biotechnology Information-Gene Expression Omnibus (GEO). The data reduction strategies narrowed down the target miRNA list and promoted the accuracy of high-throughput technology and bioinformatics. Using these data reduction strategy, we narrowed down the miRNAs list, showing that miR-29a/c, miR-34b/c, miR-429, miR-203, miR-222, miR-1/206, miR-141, miR-18a/b, miR-544, miR-205 and miR-149 may be the most important modulator during the development of NPC. The expressions of these miRNAs were also validated using real-time RT-PCR.

It is estimated that 1-4% of genes in the human genome encode miRNAs and a single miRNA can regulate as many as 200 mRNAs [49]. The expression of miRNAs can be activated or repressed by transcription factors (TFs), which therefore can serve as upstream regulators of miRNAs. In recent years, many researchers have attempted to understand how miRNAs act to regulate target genes and what their roles are in various diseases. However, the study of miRNAs regulation by TFs (TF-miRNA regulation) has been relatively limited. We reported previously that miRNAs and TFs may cooperate to regulate target gene expression. In addition, miRNAs and TFs can form feedback or feed-forward loops, which play critical roles in various biological processes. For example, ETS2 induces expression of miR-7 which, in turn, suppresses the expression of ETS2 activity. Increasing evidence suggests that aberrant regulation of miRNAs by TFs can cause diseases. Therefore, TF-miRNA regulation is one of the most important aspects of the study of both miRNAs and TFs. In this study, we bioinformatically predicted twenty-one TFs, seven of which may be the key regulator of the miRNA expression. This study provides an initial valuable data set for the miRNA regulation and its function.

## Conclusions

The goal of profiling miRNA expression in this study is to discover the specific miRNAs in which influence the development of NPC. We found that miR-29a/c, miR-34b, miR-34c-3p, miR-34c-5p, miR-429, miR-203, miR-222, miR-1/206, miR-141, miR-18a/b, miR-544, miR-205 and miR-149 may play important roles in this respect. Merging the mRNA expression array and building an integrated molecular net-work associated with NPC enables us to gain a better understanding of the overall pathology. The transcription factors that including ETS2, MYB, Sp1, KLF6, NFE2, PCBP1 and TMEM54 exert regulatory functions on the miRNA expression. Three of them were predicted to repress their own translation through a TF-miRNAs-gene feedback loop. Bioinformatics and data filtering strategy were combined to screen the miRNAs which may be the key modulator in the process of tumorigenesis.

## Competing interests

The authors declare that they have no competing interests.

## Authors' contributions

The work presented here was carried out as a collaboration between all authors. ZHL, LYZ and ZL carried out most experiments. HBY and XFG collected the biopies and the data. JJX, ZHL, ZL and GYL made contributions to design, analyze data and interpret data. JJX, ZHL, GL and XYL have been involved in drafting the manuscript. GYL gave most financial support. YFD and CJ collected and assembled the data. All the authors have given final approval to publish the manuscript.

## Pre-publication history

The pre-publication history for this paper can be accessed here:

http://www.biomedcentral.com/1755-8794/5/3/prepub

## Supplementary Material

Additional file 1 **Clinical features of patients**. The information is available online. Click here for file
